# Feasibility of a Green Breast Surgery Protocol to Reduce Carbon Footprint of Care: BuGS Trial Interim Results

**DOI:** 10.3390/jcm14196881

**Published:** 2025-09-28

**Authors:** Gianluca Vanni, Marco Materazzo, Marco Pellicciaro, Jonathan Caspi, Giordana Di Mauro, Federico Tacconi, Denisa Eskiu, Benedetto Longo, Valerio Cervelli, Massimiliano Berretta, Oreste Claudio Buonomo, on behalf of BuGS Study Group

**Affiliations:** 1 Breast Unit Policlinico Tor Vergata, Department of Surgical Science, Tor Vergata University, 00133 Rome, Italy; vanni_gianluca@yahoo.it (G.V.); marco.pellicciaro@ptvonline.it (M.P.); caspijonathan@gmail.com (J.C.); o.buonomo@inwind.it (O.C.B.); 2 PhD Program in Applied Medical-Surgical Sciences, Department of Surgical Science, Tor Vergata University, 00133 Rome, Italy; d.eskiu@prof.unizm.al; 3 School of Specialization in Medical Oncology, Department of Human Pathology “G. Barresi”, University of Messina, 98125 Messina, Italy; giordana.di.mauro@hotmail.com; 4 Unit of Thoracic Surgery, Department of Surgical Sciences, Tor Vergata University, 00133 Rome, Italy; federico.tacconi@ptvonline.it; 5 Faculty of Medicine, Università Cattolica Nostra Signora Del Buon Consiglio, 1000 Tirana, Albania; 6 Plastic Surgery, Department of Surgical Sciences, University of Rome “Tor Vergata”, 00133 Rome, Italy; benedetto.longo@uniroma2.it (B.L.); valeriocervelli@virgilio.it (V.C.); 7 Division of Medical Oncology, AOU “G.Martino” Hospital, University of Messina, 98166 Messina, Italy; berrettama@gmail.com; 8 Department of Clinical and Experimental Medicine, University of Messina, 98166 Messina, Italy; 9 General Surgery Program, Department of Health Science, University of Basilicata (UNIBAS), 85100 Potenza, Italy; 10 Department of Surgery, Sapienza University of Rome, 00133 Rome, Italy; 11 UOSD Nephrology and Dialysis, Department of Systems Medicine, Tor Vergata University, 00133 Rome, Italy; 12 Department of Diagnostic Imaging and Interventional Radiology, Molecular Imaging and Radiotherapy, Tor Vergata University, 00133 Rome, Italy; 13 Medical Oncology Unit, Tor Vergata University Hospital, 00133 Rome, Italy; 14 Department of Experimental Medicine, Tor Vergata Oncoscience Research (TOR), University of Rome Tor Vergata, 00133 Rome, Italy; 15 Department of Clinical Science and Translational Medicine, Tor Vergata University, 00133 Rome, Italy

**Keywords:** awake breast surgery, enhanced recovery after surgery, breast conserving surgery, early breast cancer, opioid-free anesthesia, telemedicine, sustainable surgery, carbon footprint, randomized controlled trial, postoperative pain

## Abstract

**Background/Objectives**: Our objective was to evaluate the clinical feasibility and sustainability of an awake breast conserving surgery (BCS) protocol integrating opioid-free anesthesia, telemedicine, and environmental sustainability compared to standard care. **Methods**: A prospective, monocentric, non-inferiority randomized controlled trial (133.24 CET2 ptv; NCT06624917) named Breast Green Surgery (BuGS) was planned. Women aged 18–75 years eligible for level I BCS were randomized to either the BuGS group—comprising opioid-free monitored anesthesia care (MAC), a telehealth follow-up, and intraoperative waste reduction—or the control. The primary endpoint was postoperative pain at rest (PPR) and during movement (PPD) both at 24 h, measured with the Numeric Pain Rating Scale (NPRS). Secondary exploratory endpoints included SF-36, PSQ-18, TSQ test, LOS, and KgCO_2_ equivalent (KgCO_2_e) from surgical waste, patient transport, and complications. The interim analysis included patients completing a 30-day follow-up by 31 December 2024. **Results**: A total of 45 patients were enrolled (BuGS n = 18, control n = 27). While disproportionate, no significant differences were observed in PPR/PPD at 24 h (4.75 (3.725–5.875) vs. 4.5 (4.15–5.35); *p* = 0.626; 4 (3.10–4.75) vs. 4.6 (3.90–5.2); *p* = 0.130), confirmed using group ANOVA analysis (*p* = 0.515; *p* = 0.779, respectively). The BuGS group reported a reduced surgical room occupation (80.03 (64.84–101.87) vs. 133.23 (95.47–144.25) min; *p* = 0.002) and length of stay (0 (0–1) vs. 1 (1–2); *p* = 0.0001), without hospital unplanned admissions. Reduced KgCO_2_e emissions from waste disposal were reported, with no difference in SF-36, PSQ-18, and complication rates. **Conclusions**: If confirmed after complete accrual, BuGS could potentially promote a clinically equivalent, environmentally sustainable, and hospital efficient surgery without affecting the QoL of our patients. Further multicentric validation is warranted.

## 1. Introduction

Breast cancer (BC) is the most common oncological diagnosis in the female population, and its incidence is expected to increase over the next 20 years, requiring healthcare resource optimization in the near future [1].

Breast conserving surgery (BCS), when feasible, is considered the gold standard procedure, especially in early BC (EBC) care, allowing a broader implementation of awake breast surgery protocols [2,3]. The awake BCS protocol defines an opioid-free (OF) monitored anesthesia care (MAC) approach, utilizing local anesthesia, inter-fascial plane blocks, and sedation. In awake BCS, the surgical procedure is carried out without mechanical ventilation. Previous analyses demonstrated that awake BCS could promote enhanced recovery after surgery, reduce the psychological impact, and attenuate the postoperative stress response associated with surgery-induced immunosuppression [4,5].

While routinely applied, the awake BCS rate in our clinical facilities increased after the COVID-19 outbreak, ensuring a steady activity during the lockdown period [6]. In the same period, reduced hospital admissions were achieved through telehealth assessments in our outpatient clinical practice. All these measures contributed to a reduction in length of stay (LOS) and hospital admissions, as well as a reduction in potential hospital cross-infection. However, COVID-19 restrictive measures had an unintended benefit in lowering healthcare-related environmental pollutants [7,8]. Indeed, lockdown policies and reduced hospital admissions were responsible for positive environmental effects on a worldwide scale [9,10].

The healthcare sector is both burdened by, and actively contributes to, environmental pollution, an issue that is not always familiar to healthcare providers [11]. Healthcare facilities are responsible for up to 10% of greenhouse gas emissions (GHG) in Western countries. Operating rooms currently make a significant contribution to the healthcare carbon footprint [12,13]. Although awake BCS was routinely applied during the COVID-19 pandemic, no formal analysis has been conducted to assess its impact on clinical outcomes, patients’ reported outcome measures (PROM), or environmental reported outcome measures (EROM) to evaluate its clinical applicability beyond the pandemic emergency [14]. Therefore, a randomized controlled trial, named a Breast Green Surgery (BuGS) trial, was planned in our institution.

## 2. Materials and Methods

### 2.1. Study Design

The BuGS trial is designed as a prospective, monocentric, non-inferiority randomized clinical trial (RCT). The primary endpoint of the study was defined as postoperative pain at rest (PPR) and postoperative pain dynamic (PPD) assessed 24 h after surgery. Secondary outcomes of the study included clinical measures such as 48 h and 30-day PPR and PPD; PROMs assessing telehealth implementation via the Telemedicine Satisfaction Questionnaire (TSQ); and Quality of Life (QoL) evaluated using the Short-Form Patient Satisfaction Questionnaire (PSQ-18) and the Short-Form 36 Health Survey (SF-36). Finally, EROM was assessed through metrics of hospitalization days, number of hospital admissions after surgery, operating room carbon footprint, waste recycling, and reduced admissions in the hospital. While PPD and PPR were considered the primary outcomes, other outcomes were analyzed as secondary or exploratory. No formal adjustment for multiple testing was performed; therefore, secondary findings should be interpreted with caution.

The study protocol was approved by the local Ethics Committee of Lazio Area 2 (133.24 CET2 ptv) and registered at clinicaltrial.gov NCT06624917. Hence, participant recruitment began on 2 September 2024, and the study enrolment is currently ongoing. This manuscript includes an interim analysis of the participants who completed follow-up by 31 December 2024. Data were analyzed between 1 January 2025 and 1 February 2025. The first three months of the study (cutoff: 31 December) were analyzed to provide preliminary safety and feasibility data while the trial was ongoing. This manuscript follows the Consolidated Standards of Reporting Trials (CONSORT) reporting guideline for randomized clinical trials [15].

### 2.2. Study Population

Eligible patients were female candidates for level I BCS between the ages of 18–75, with an American Society of Anesthesiologists (ASA) score I-II and preliminary availability for telehealth assessment during the postoperative period. Exclusion criteria included drug addiction, contraindications for a locoregional ultrasound-guided procedure (e.g., local infection, allergy to local anesthetics (LA)), chronic pain under treatment, pregnancy, and lack of planned follow-up at our facility. All patients were required to complete a preoperative assessment and were enrolled in the study at the time of the preoperative visit.

For sample size calculations, 24 h PPR and PPD were calculated from a preliminary analysis of a pilot study involving 10 participants in each group, comparing patients undergoing BuGS surgery (BuGs group) and a control group. Based on data from a prior study conducted by our group, the effect size was calculated as 0.139, with a standard error of 0.234. Alpha and Beta errors were set at 0.05 and 0.10, respectively, and Δ was set to 1. Therefore, the sample size was calculated as 49 participants in each group. Calculating a drop-out rate of 10%, enrollment was set at 110 participants.

### 2.3. Randomization and Treatment

#### 2.3.1. Randomization

Once enrolled, written informed consent was obtained from the patients. Participants were randomized into the BuGS group or the control group, and block randomization was performed using Excel (Microsoft, Redmond, WA, USA). After enrollment, home address and patients’ transportation modes were registered for any new hospital admission. During the initial admission, all participants received instructions on telehealth assessment using Teams Software 1.6 (Microsoft, Redmond, WA, USA).

**Figure 1:**
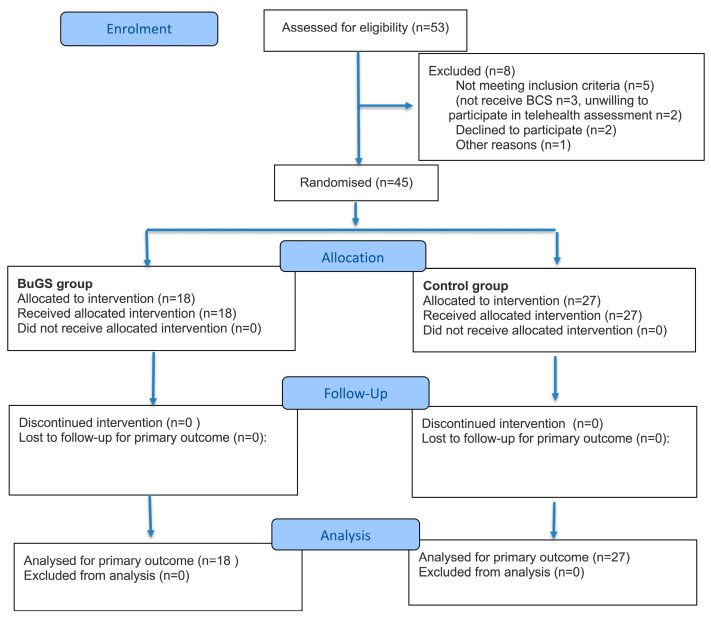
Consort flow diagram.

Participants and healthcare workers were blinded to group allocation through a compartmentalized blinding strategy, whereby each professional was aware only of the information required for their role. Allocation concealment was ensured through sealed opaque envelopes, which were opened during care by the physician involved. Postoperative assessment and telephonic follow-up were carried out by a physician unaware of group allocation (Figure 1).

#### 2.3.2. BuGS Group

The BuGS protocol was designed by merging our previous experience with awake breast surgery, enhanced recovery after surgery (ERAS) protocol already published in breast cancer care, telehealth assessment, and Intercollegiate Green Theatre Checklist (Table A1) [4,16,17]. Patients enrolled in the BuGS group were admitted on the day of surgery through the outpatient clinic to avoid hospital bed occupancy. Furthermore, morning scheduling of all BCS procedures was implemented to reduce avoidable hospital bed occupancy. After admission to the surgical facilities, preoperative assessment and multimodal pain management, including tailored antibiotic prophylaxis and target inter-fascial plane blocks when clinically appropriate, were utilized to reduce the need for systemic treatment. Approved anesthesia regimens within the research protocol included LA or awake breast surgery protocol, consisting of an opioid-free MAC with or without mild sedation with a Richmond agitation–sedation scale target (RAS) > −3 and <1, and without use of airway management devices. Different levels of sedation and MAC were permitted as long as mechanical ventilation was not required. Our intentional approach in the study was designed to simulate real-world clinical practice, while introducing some heterogeneity.

During the procedure, the operating room was arranged to optimize waste reduction and segregation. Monitoring the number of staff members in the surgical room aimed to reduce waste production. The reduction in single-use surgical instruments and other consumables was intended to reduce non-sterile glove usage, except when in contact with mucous membranes, biological fluids, or wounds. The BCS instrument set was carefully designed to reduce waste production and decrease the number of unused instruments during surgical procedures. Three different instrument sets were prepared: the Essential, Complete, and Oncoplastic kits. All three instrument sets were ready for use in the operating room, but were not opened unless specifically requested.

During the procedures, surgical room waste segregation and recycling were encouraged to reduce the carbon footprint. At the end of the surgical procedure, all medical waste (MW) was classified as contaminated medical waste (CMW), non-contaminated medical waste (NCMW), plastic medical waste (PlMW), and paper medical waste (PaMW).

Patients remained in the outpatient clinic for 3 h after surgery and were re-evaluated to assess whether hospitalization was needed; if not, they were discharged. Patients were discharged if clinical conditions were deemed appropriate by the surgeon and anesthesiologist (effective pain control, stable vital signs, ability to tolerate oral intake, spontaneous diuresis, independent ambulation, and absence of immediate postoperative complications). Postoperative assessment ensured that patients live within a 1 h drive from the hospital and had a responsible caregiver available during the first 24 h. After discharge, postoperative assessment was carried out through telehealth assessment via Teams Software 7 days after surgery. If requested by patients and/or healthcare providers, hospital admission was arranged. Regardless, suture removal was planned for 12 days postoperatively, either through hospital admission or a GP appointment, according to the participants’ preference.

#### 2.3.3. Control Group

The control group was admitted according to the standard of care (SoC) of our hospital (Table A1). Admission was scheduled for the same day of surgery, or one day prior if any preoperative assessment was required (e.g., preoperative examination, or preoperative procedure as reperage). Participants were admitted to the hospital ward. Upon admission, preoperative assessments were carried out to plan the surgical procedure and determine the preferred anesthetic regimen, with options including LA, inter-fascial plane block, awake breast surgery, Volatile General Anesthesia (VGA), and Total Intravenous Anesthesia (TIVA). Moreover, in the control group, the use of airway management devices (such as laryngeal masks or orotracheal intubation) was permitted, but not mandatory, and was left to the discretion of the anesthesiologist based on clinical judgment. In the control group, neither waste reduction nor waste segregation were implemented. No limitations were placed on surgical instruments, which were prepared according to the surgeon’s preference. During surgical procedures, operating room waste segregation and recycling were not actively encouraged, although MW was sub-classified as in the BuGS group (CMW, NCMW, PlMW, PaMW). After surgery, all patients were admitted to the surgical department and discharged in the following days according to their clinical condition. Postoperative assessment was always carried out in the outpatient clinic, according to the SoC, and hospital admission for suture removal was scheduled 12 days after surgery.

### 2.4. Data Collection

#### 2.4.1. Demographic Data and Clinical Outcome

All demographic and clinical data, such as age, Body Mass Index (BMI), smoking status, combined estrogen–progestin therapy, and preoperative diagnosis (when available), were collected from clinical records, in accordance with the guidelines for non-operative diagnostic procedures and reporting in breast cancer screening by the Royal College of Pathologists [18]. Preoperative comorbidities were assessed using the Charlson Comorbidity Index (CCI) after enrollment. After surgery, PPR and PPD were collected from patients at the end of the procedure, and at 2 h, 24 h, and 48 h postoperatively, using the Numeric Pain Rating Scale (NPRS) with telephonic follow-up or during hospitalization. At 30 days, PPR, PPD, and Clavien Dindò complication evaluation were retrieved through telephonic follow-ups and collected from clinical notes [19]. Moreover, hospitalization rates and LOS were assessed in both groups. LOS was defined as 0 for patients whose surgical care was completed in the outpatient clinic.

#### 2.4.2. Environmental Outcome

Environmental impact was estimated in terms of KgCO_2_e emissions, based on indirect evaluation and calculated according to the literature data. This simplified approach was designed to provide a feasible and reproducible estimate, while acknowledging that it does not replace a comprehensive life-cycle analysis.

According to a previous analysis, private car transportation was the sole means by which our patients traveled to the hospital. Private car transportation pollution was calculated in kilograms of carbon dioxide equivalent (KgCO_2_e). The distance was considered to be twice the one-way distance between a participant’s home and the hospital. Mean private car fuel consumption was calculated as 11 L per 100 km, with an average emission factor of 2.34 KgCO_2_ per liter, as reported in a previous similar analysis [20]. Therefore, KgCO_2_e was calculated as follows:

$${Kg}_{{Co}_{2}e}=2.34\frac{11}{100}(Distance)$$

The total number of admissions across the entire study period was included in the analysis to determine the KgCO_2_e associated with transportation. Moreover, workday losses for both participants and caregivers were calculated over the entire study period.

Intraoperative assessment included surgical room occupancy, surgical procedure duration in minutes, and the number of instrument kits opened during surgery. MAC was defined as local, locoregional, TIVA, or VGA. Waste segregation included Kg of CMW, NCMW, PlMW, and PaMW produced during each procedure. Waste disposal impact was calculated as KgCO_2_e produced for each CMW and NCMW. For CMW, incineration remains the predominant method of hospital waste disposal, primarily due to the potentially infectious nature of such waste. The KgCO_2_e per kg of CMW was calculated as follows [21]:

$${CMV\;Kg}_{{Co}_{2}e}=1.074\;{Kg}_{CMV}$$

For NCMW, landfilling is the predominant method, and its associated KgCO_2_e produced was calculated by Manfredi et al. as follows [22]:

$${NCMV\;Kg}_{{Co}_{2}e}=0.300\;{Kg}_{NCMV}$$

The first analysis compared landfilling impact between groups (BuGS vs. control). Moreover, a second analysis was then performed to include the impact of recycling and second raw material production.

In the control group, no waste segregation was encouraged, and the cost of secondary raw material production of PlMW and PaMW was included in the analysis, in comparison with the BuGS group’s waste disposal and recycle costs. PlMW and PaMW sent to landfill were recorded in kilograms and included in the analysis. Secondary raw material production emissions were calculated according to the UK Department for Environment, Food & Rural Affairs (DEFRA), Institute for Applied Ecology, and European Commission Joint Research Centre data [22,23,24,25]:

$$\begin{array}{ll} {NCMV\;Kg}_{{Co}_{2}e}= & NCMVwaste\;landfilling\\ & +\;Paper\;secondary\;material\;production\\ & +\;Plastic\;secondary\;raw\;material\;production\\ & =0.300\;{Kg}_{NCMV}\;+\;{0.910\;Kg}_{PaMV}\;+\;{3.0\;Kg}_{PlMV} \end{array}$$

In the BuGS group, PlMW and PaMW waste disposal was calculated as the sum of landfill emissions for unrecycled material plus recycle cost, and was compared to the NCMW related KgCO_2_e in the control group as follows [22,23,24,25]:

$$\begin{array}{ll} {NCMV\;Kg}_{{Co}_{2}e}= & NCMVwaste\;landfilling\;+\;Paper\;reciclying\\ & +\;Plastic\;secondary\;reciclying\\ & =\;0.300\;({Kg}_{NCMV}\;-\;{Kg}_{PaMV}\;-\;{Kg}_{PlMV})\;+\;{0.730\;Kg}_{PaMV}\\ & +\;{0.311\;Kg}_{PlMV} \end{array}$$

#### 2.4.3. Quality of Life and Satisfaction Assessment

QoL was assessed using the TSQ in the BuGS group and control group with SF-36 and PSQ-18 at 30 days postoperatively via telephone follow-up. Generic PROM scores were intentionally selected to ensure reproducibility and allow potential scalability of the trial across different surgical specialties.

TSQ is a validated 14-item questionnaire developed in 2003 to evaluate patients’ experience with telemedicine [26,27]. Firstly evaluated in diabetic patients, this score has since been proposed for assessing the quality of telehealth care in oncological patients and breast patients [26,27]. The questionnaire is composed of three main components regarding quality of care provided (TSQ1), similarity to an outpatient visit (TSQ2), and perception of the interaction (TSQ3). TSQ uses a 5-point Likert scale (“Strongly disagree” (1) to “Strongly agree” (5)). The total TSQ score ranges from 14 to 70 with different values between subsets: TSQ1 from 8 to 40, TSQ2 from 5 to 25, and TSQ3 from 1 to 5. A total score >56 has been considered indicative of a good patient experience.

SF-36 is a questionnaire used to evaluate health-related QoL, with 36 questions that are divided into eight domain profiles of score: physical functioning (PF), 10 items; general health (GH), 5 items; role physical (RP), 4 items; bodily pain (BP), 2 items; social functioning (SF), 2 items; vitality (VT), 4 items; role emotional (RE), 3 items; and mental health (MH), 5 items. Each domain score ranges from 0 to 100 (higher score is associated with better health-related QoL) [28].

PSQ-18 was developed in 1994 as a short form of the PSQ-50 to evaluate patients’ satisfaction [29]. PSQ-18 includes seven subscales, each correlating with seven different aspects of satisfaction with medical care: general satisfaction, technical quality, interpersonal manner, communication, financial aspects, time spent with doctor, and accessibility and convenience. All 18 items are evaluated using a 5-point Likert scale, with higher scores indicating greater satisfaction. In this analysis, the mean PSQ-18 score across all items was included in the study.

### 2.5. Statistical Analysis

Data were recorded in an EXCEL database (Microsoft, Redmond, WA, USA). Continuous variables are reported as medians with interquartile ranges, and were analyzed by the Wilcoxon–Mann–Whitney test. The Chi-squared test (or Fisher’s exact test, depending on group size) was used to analyze categorical dichotomous variables. If no dichotomous variables were present, the Monte Carlo test adjustment was applied. Repeated measures such as PPR and PPD were assessed with two-way repeated measures ANOVA to evaluate time effect, time × group effect, and between-group effect. All variables with a *p*-value of <0.05 were considered statistically significant. Statistical analyses were performed using the SPSS statistical package version 26.0 (SPSS Inc., Chicago, IL, USA).

## 3. Results

From 2 September to 30 November 2024, a total of 53 patients were assessed for eligibility. Of these, eight patients were excluded from the study, and five did not meet the inclusion criteria: three patients did not undergo BCS, two patients were unwilling to participate in telehealth assessment, two patients declined to participate in the study, and one patient decided to not have a follow-up at our outpatient clinic. Therefore, 45 patients were included in the study sample and were randomized between the BuGS group (n = 18) and the control group (n = 27), as reported in Figure 1.

Preoperative demographic data were analyzed between groups, and no differences were reported. There were no statistically significant differences reported in age, menopausal status, combined estrogen–progestin therapy, and comorbidities reported as CCI (*p* > 0.05). Similar rates of BC (B5) diagnosis were reported between groups (3 (16.7%) vs. 11 (40.7%); *p* = 0.393). No difference in hospital distance was reported between groups, although the BuGS group lived farther from the hospital compared to the control group (21.50 vs. 12.00 km, *p* = 0.365) (Table 1).

Intraoperative and postoperative characteristics are listed in Table 2. The BuGS group exhibited a reduced surgical room occupation (80.03 (64.84–101.87) vs. 133.23 (95.47–144.25) minutes; *p* = 0.0002); also, the surgical procedure duration was shorter compared with the control group (44.72 (24.64–61.56) vs. 87.23 (44.68–91.34) minutes; *p* = 0.0003). Moreover, a reduced use of instrument kits was observed in the BuGS group (*p* = 0.003). In the control group, two different instrument kits were always opened, while, in four (22.3%) cases in the BuGS group, the oncoplastic adjunctive kit was not deemed necessary by the surgeon. The MAC strategy demonstrated a higher rate of locoregional and local anesthesia in the BuGS group when compared with the control group (*p* < 0.0001). The awake breast surgery protocol, using LA or locoregional anesthesia, was successfully carried out in all patients in the BuGS group, with zero (0%) conversions reported. In the control group, only three (11.1%) patients underwent awake breast surgery. The preferred MAC regimen was TIVA, with 21 (77.8%) participants in the control group.

Intraoperative waste segregation demonstrated a reduced amount of CMW (3.09 (2.86–3.26) vs. 3.95 (3.4–4.38) kg; *p* < 0.0001) and NCMW (0.43 (0.38–0.49) vs. 0.94 (0.86–1.04) kg; *p* < 0.0001) between groups. The reduced amount of CMW consequentially determined a reduced KgCO_2_e for its disposal in the BuGS study compared to the control group (3.31 (3.07–3.51) vs. 4.24 (3.65–4.69) KgCO_2_e; *p* < 0.0001). Additionally, a “Don’t open it unless you need it” approach determined a reduced KgCO_2_e amount from landfilling (0.13 (0.11–0.15) vs. 0.28 (0.26–0.31) KgCO_2_e; *p* < 0.0001). An even greater reduction in GHG emissions was observed when recycling and secondary raw material production were included in the analysis (0.19 (10.16–0.21) vs. 2.15 (2.07–2–44) KgCO_2_e; *p* < 0.0001).

**Figure 2:**
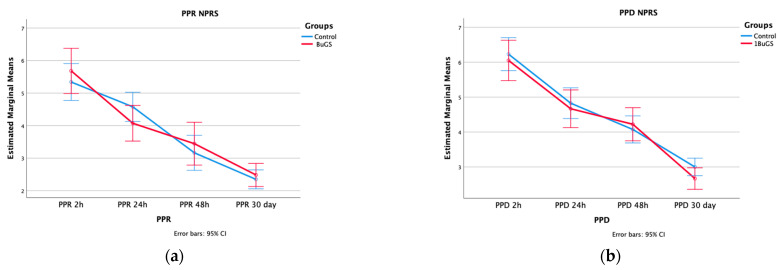
PPR and PPD analysis. (**a**) PPR according to the study period; (**b**) PPD according to the study period; all data are reported as median and 95% CI.

Postoperative assessment included PPR and PPD at 2 h, 24 h, 48 h, and 30 days. As the primary outcome, no statistically significant differences were found between groups in PPR (4 (3.10–4.75) vs. 4.6 (3.90–5.2); *p* = 0.130) and PPD (4.75 (3.725–5.875) vs. 4.5 (4.15–5.35); *p* = 0.626). Regarding PPR, a steady decrease was reported across groups. Repeated measures ANOVA showed a significant effect of time on PPR (*p* < 0.001), but no significant time × group interaction, with no difference in NPRS across time (*p* = 0.287). Moreover, no between-group effect was observed (*p* = 0.779). Similarly, a decrease was reported in PPD. PPD, as expected, showed a time-related decrease across groups (*p* < 0.001), with no significant time × group interaction difference in NPRS (*p* = 0.534), and no between-group statistically significant difference (*p* = 0.515) (Figure 2).

Hospitalization occurred in nine (50.00%) participants in the BuGS group, due to a lack of oral intake after surgery. Despite a high rate of unplanned hospitalization, the BuGS protocol resulted in a reduced LOS compared to the control group (0 (0–1) vs. 1 (1–2); *p* = 0.001). Hospital admissions following telehealth assessments were lower in the BuGS group (1 (1–1.75) vs. 2 (2–2); *p* = 0.0002), while KgCO_2_e related to patient transportation by car to the hospital was comparable between groups (7.26 vs. 6.07 KgCO_2_e; *p* = 0.933). A reduced number of work absences was recorded in the BuGS group (*p* = 0.001). At 30 days, Clavien Dindò complications of grade >2 failed to demonstrate a statistically significant difference (1 (5.6%) vs. 2 (7.4%); *p* = 0.615). Among surgical complications, all three patients were treated in the outpatient’s facility: two patients required fine-needle aspiration for a postoperative seroma, and one participant received outpatient drainage packing due to a surgical site infection. Additionally, no unplanned surgical re-admissions were recorded at 30 days follow-up (Table 3).

Finally, QoL assessment demonstrated a lack of statistical differences in SF-36 and PSQ- 18, as reported in Table 4. TSQ in the BuGS group reported a high grade of satisfaction among BuGS patients; the median value was 43.00, and 12 (63.26%) participants gave a score higher than 40 (Table 4).

## 4. Discussion

While still at interim analysis, the initial data from the BuGS randomized clinical trial demonstrates that the BuGS protocol in BCS can be considered safe in terms of PPR and PPD, without detrimental effects on patients QoL according to the SF-36 and PSQ-18. Furthermore, the results suggest that telehealth assessment is well accepted by patients and, when integrated into an enhanced recovery pathway, may provide beneficial effects on hospitalization rates, healthcare costs, and the carbon footprint of care.

Modern BC care aims for a safe de-escalation of treatment, considering the growing number of patients, especially in EBC. Shubeck et al. described the low value of invasive surgical procedures which do not improve oncologic or survival outcomes and do not provide clinical, economic, or social cost benefits. Short- and long-term surgical complications, further low-value medical procedures, increased expenses related to travel for patients and caregivers, direct and indirect costs, and patients’ time costs contribute to excessive healthcare utilization and system saturation [30].

EBC is now widely recognized as an area suitable for safe surgical de-escalation, thanks to the early diagnosis campaigns and large, high-quality randomized clinical trials that have demonstrated an overtreatment risk in many patients. In the past decade, the “no ink on tumor” guideline was formalized, leading to a reduction in specimen resection volumes and a reduction in reoperation rates [31,32]. Additionally, the AMAROS and Z0011 trials provided an evidence-based framework ensuring a safe and effective management in patients with low axillary burden [33,34]. More recently, SLNB were not considered mandatory in axillary US negative EBC and in elderly patients, as reported in the 2025 SLNB ASCO guidelines [35,36,37]. Moreover, multidisciplinary de-escalation strategies, such as omitting adjuvant radiotherapy in low risk elderly patients with EBC, are considered acceptable, thus reducing healthcare system saturation [38]. Within this perspective, surgical de-escalation in EBC eases safe outpatient management, maximizing ERAS protocols and awake surgery benefits in terms of postoperative recovery and hospitalization [4,17]. ERAS and awake protocol have been designed to attenuate postoperative stress, optimize immunological function, and improve recovery by multimodal OF analgesic strategies [39]. In fact, in small–medium surgery, such as BCS, OF MAC improves postoperative recovery, limiting postoperative nausea and vomiting and maintaining a stable hemodynamics throughout the surgical procedure [40].

Besides its effect on the early postoperative period, many authors investigated potential benefits for long-term oncological outcomes [39,41]. TIVA and opioids have been associated with a greater immunosuppression when compared with TIVA or regional techniques, which seem to maintain a steady immune function and natural cell activity and overall survival [41,42]. Classically, surgical trauma induces significant neuroendocrine signaling that affects metabolic processes and immune and inflammatory pathways, potentially promoting epithelial–mesenchymal transition (EMT), angiogenesis, and tumor cell spreading [43], and perioperative measures should be taken into account to reduce postoperative neuro-inflammation, which has been closely associated with postoperative pain, chronic postoperative pain, and surgical complications [44,45,46]. While perioperative immunomodulation is still an active research field, a recent meta-analysis showed that BCS plus adjuvant treatment provided better oncological outcomes when compared with mastectomy in EBC [47]. Thanks to these results, modern clinical practice encourages BCS in carefully selected hereditary BC patients and for patients requiring oncoplastic procedures with extensive glandular reshaping, thus increasing the number of patients undergoing BCS in the future and potentially transitioning to awake BCS [48,49].

Applying surgical de-escalation in this setting, while clinical effective, may determine a novel interest toward the reduction in perioperative care needs, hospitalization, and carbon footprint of care without affecting QoL in our patients. As expected, our study population did not exhibit statistical differences in postoperative PPR, PPD, and postoperative complication rates.

In our clinical settings, BuGS patients demonstrated a reduction in surgical room occupancy and surgical procedure time. From a hospital perspective, 70% of the total GHG produced arises from operating rooms, with a single operation ranging from 6 kg and 814 KgCO_2_e, equivalent to an average private car traveling 2 273 miles [13,50]. Additionally, in the BuGS protocol, all patients successfully underwent MAC, with a significant effect on sustainability. Moreover, the anesthetic regimen is a neglected source of pollution and GHG emissions. When compared to local anesthesia, locoregional techniques, or TIVA, GVA demonstrated a higher impact on the environment. For instance, a single Desflurane vaporized bottle could produce 886 KgCO_2_e [16,51]. Lastly, multidisciplinary waste minimization (“Don’t open it unless you need it”) contributed to a reduction in CMW and NCMW, and showed a statistically significant difference in terms of KgCO_2_e emissions, even larger when integrated with the secondary production of raw materials.

Hospital waste and healthcare pollutants are strongly related to health and environmental determinants through an accelerating spillover effect, increasing the exposure to carcinogenic agents related to climate change, changes in healthy behavior, and an increase in natural disasters that may impair elective and preventive resource allocation, including for oncological diseases. Therefore, de-escalating the environmental impact of healthcare may contribute positively to global health outcomes [14]. Additionally, synergistic effects of reduced surgical room occupancy, reduced hospitalization, and reduced CMW and NCMW could bring about a decrease in hospital costs and service saturation. Previous evidence demonstrated how ERAS protocols in breast surgery could provide beneficial effects by increasing bed capacity and surgical volume, without changes in hospital charges [52].

Further benefits are associated with the reduction in the surgical BC waiting list. Accelerated recovery protocols can effectively reduce the number of patients treated more than eight weeks after diagnosis, a well-known factor associated with BC-related mortality [53,54]. Our protocol was further designed to reduce as many hospital admissions after surgery as possible and to decrease outpatient clinic saturation. Telehealth assessment was activated during the COVID-19 pandemic at our institution, and it continues to be integrated within our BuGS protocol. Participants demonstrated a high satisfaction in BC telehealth and telehealth assessment showed a reduction in BuGS hospital admissions, though KgCO_2_e car pollution failed to demonstrate a statistically significant difference between groups. While preliminary, our data demonstrate how the BuGS protocol is not perceived as inferior to the standard of care.

However, some limitations should be disclosed in this study. Firstly, our preliminary data need to be confirmed in the final analysis of the study; yet, as reported in several studies, PPR and PPD pain control and reduced hospitalization were comparable to previous analyses conducted by our group before and after the COVID-19 pandemic [6,55]. Additionally, the reduced statistical power inherent to the interim analysis and unbalanced groups means that our results should be considered initial and that definitive conclusions should be deferred until full accrual and final analysis. Furthermore, secondary outcomes were considered exploratory, and no sample size analyses were performed or adjustments made. However, while initial, our analysis demonstrated how innovative perioperative management may not affect postoperative outcomes and may effectively reduce carbon footprints.

Second, the monocentric design may limit the external applicability of our data regarding the reduction in KgCO_2_e emissions. Additionally, all the environmental outcomes were derived from a KgCO_2_e estimation and not from an LCA. This choice was dictated by feasibility in the initial EROM assessment. Future analyses are planned to incorporate such methodology. However, many initiatives have been designed and have independently demonstrated a reduction in the carbon footprint of care, such as Wide Awake Local Anesthesia No Tourniquet (WALANT) hand surgery [56]. Another limitation of the study design is the restrictive selection of patients, which introduces a potential selection bias and reduces the applicability of the study. While most of the patients in BC surgery fall into study age range, the selection of healthy subjects was deliberate to ensure patient safety and a homogeneous population. Once accrual is complete and the final analysis is available, further studies will incorporate a larger population (e.g., elderly, ASA > III) for whom awake BCS has resulted in reduced perioperative morbidity [57]. This future research will enable population stratification and clarify which subgroups derive the greatest benefit from this perioperative pathway.

Additionally, the BuGS protocol included variable sedation levels and different non-mechanical ventilation MAC regimens that could increase group heterogeneity. However, this planned assessment was intended to capture real-world practice, where different regimens could be required depending on patient tumor locations and tumor-to-breast ratios.

Third, although no detrimental effects on QoL or increases in complication rates were observed, the current short follow-up period may not be sufficient to capture long-term outcomes. However, while no clear detrimental effects have been recorded in the long-term in the literature to our knowledge, the benefits from ERAS pathways, as well as from OF awake surgery, include faster recovery, faster systemic adjuvant administration, and better long-term oncological outcomes [43,58]. Another limitation of QoL assessment is the use of generic PROMs. While these instruments were intentionally chosen to ensure reproducibility and scalability across different surgical specialties, they may lack the disease-specific sensitivity of breast-focused tools such as the EORTC QLQ-C30 with BR45 or the BREAST-Q. Future phases of the study will therefore integrate disease-specific PROMs to provide a more comprehensive PROM assessment.

Despite these limitations, the initial data from the BuGS study demonstrate how the specific perioperative protocol may promote a faster recovery without affecting early postoperative outcomes, potentially reducing hospitalizations with a potential positive effect on the carbon footprint of BC care, without detrimental effects on patients’ perceived care. While further enrollment is needed to complete the BuGS study, if confirmed after complete accrual and through further larger multicentric prospective analyses, BuGS could potentially promote a clinically equivalent, environmentally, and hospital sustainable surgery without affecting QoL in our patients.

## Figures and Tables

**Table 1:** Demographic characteristics.

Characteristics	Groups		*p*-Value
	BuGS Group (n = 18)	Control Group (n = 27)	
**Age, yrs (IQR)**	44.18 (40.28–45.91)	46.74 (43.92–50.47)	0.122
**Menopausal status n (%)**			
**Yes**	2 (11.1%)	8 (29.6%)	0.272
**No**	16 (88.9%)	19 (71.4%)	
**BMI, Kg/m^2^ (IQR)**	23.73 (21.53–26.21)	26.04 (21.66–27.69)	0.555
**Smoke habits, yes (%)**	9 (37.5%)	15 (63.5%)	0.714
**EPT, yes (%)**	7 (58.3%)	5 (41.7%)	0.130
**CCI (IQR)**	2.50 (2–3)	2.93 (2–3)	0.09
**Distance of hospital from home, km (%)**	21.50 (7.72–29.65)	12.00 (7.9–21.8)	0.365
**Preoperative diagnosis n (%)**			
**B2**	3 (16.7%)	3 (11.1%)	0.393
**B3**	10 (55.6%)	11(40.7%)	
**B5**	3 (16.7%)	11(40.7%)	
**NA**	2 (11.1%)	2(7.4%)	

Continuous variables are reported as median and IQR. Categorical variables are reported as frequencies and percentage. IQR: interquartile range; BMI: Body Mass Index; EPT: estrogen–progestin therapy; CCI: Charlson Comorbidity Index.

**Table 2:** Intraoperative data.

Characteristics	Groups		*p*-Value
	BuGS Group (n = 18)	Control Group (n = 27)	
**Surgical room occupation min. (IQR)**	80.03 (64.84–101.87)	133.23 (95.47–144.25)	**0.002**
**Surgical procedure min. (IQR)**	44.72 (24.64–61.56)	87.23 (44.68–91.34)	**0.0003**
**Intrument list n (%)**			
Essential	1 (5.6%)	0 (0%)	**0.003**
Complete	3 (16.7%)	0 (0%)	
Essential + Oncoplastic	13 (72.2%)	16 (59.3%)	
Complete + Oncoplastic	1 (5.5%)	11 (40.7%)	
**Anesthesia n (%)**			
LA	2 (11.1%)	0 (0%)	**<0.0001**
Locoregional	16 (88.9%)	3 (11.1%)	
TIVA	0 (0%)	21 (77.8%)	
VGA	0 (0%)	3 (11.1%)	
**Waste segregation kg (IQR)**			
CMW	3.09 (2.86–3.26)	3.95 (3.4–4.38)	**<0.0001**
NCMW	0.43 (0.38–0.49)	0.94 (0.86–1.04)	**<0.0001**
PlMW	0.25 (0.24–0.30)	-	
PaMW	0.12 (0.11–0.15)	-	
**Waste disposal, KgCO_2_e (IQR)**			
CMW	3.31 (3.07–3.51)	4.24 (3.65–4.69)	**<0.0001**
NCMW	0.13 (0.11–0.15)	0.28 (0.26–0.31)	**<0.0001**
**Waste disposal + recycling + secondary raw material production, KgCO_2_e (IQR)**			
NCMW	0.19 (10.16–0.21)	2.15 (2.07–2–44)	**<0.0001**

Continuous variables are reported as median and IQR. Categorical variables are reported as frequencies and percentage. Statistically significant *p*-values are in bold. IQR: interquartile range; LA: local anesthetic; TIVA: Total Intravenous Anesthesia; VGA: Volatile General Anesthesia; CMW: contaminated medical waste; NCMW: non-contaminated medical waste; PlMW: plastic medical waste; PaMW: paper medical waste.

**Table 3:** Postoperative data.

Characteristics	Groups			*p*-Value			
	BuGs Group (n = 18)	Control Group (n = 27)					
**Outpatient hospital admission n (IQR)**	1 (1–1.75)	2 (2–2)		**0.002**			
**KgCO_2_e car single route km (IQR)**	5.53 (1.99–7.63)	3.09 (2.033–5.61)		0.365			
**KgCO_2_e car all routes km (IQR)**	7.26 (1.99–13.35)	6.07 (4.07–11.22)		0.933			
**Sick leave**							
>1 day	1 (5.9%)	4 (23.5%)		**0.001**			
1 day	13 (76.5%)	16 (94.1%)					
0 days	4 (36.6%)	7 (63.4%)					
**Clavien Dindò complication**							
0–2	17 (94.4%)	25 (92.6%)		0.615			
>2	1 (5.6%)	2 (7.4%)					
**Postoperative Pain**			**Wilcoxon–Mann–Whitney**		**ANOVA**		
				***p*** **-Value**	**Time**	**Time × Group**	**Between-Group** **Effect**
**PPR (IQR)**							
2 h	6.2 (4.10–6.57)	5 (4.40–6.15)		0.562	**<0.001**	0.287	0.779
24 h	4 (3.10–4.75)	4.6 (3.90–5.2)		0.130			
48 h	3.10 (2.5–4.45)	3.30 (2.00–4.00)		0.907			
30 days	6.20 (4.10–6.50)	5 (4.40–6.15)		0.665			
**PPD (IQR)**							
2 h	6.2 (5.1–7.15)	6.1 (5.25–7.15)		0.618	**<0.001**	0.534	0.515
24 h	4.75 (3.725–5.875)	4.5 (4.15–5.35)		0.626			
48 h	4.00 (3.25–5.00)	4.00 (3.00–5.00)		0.628			
30 days	3.00 (2.00–3.00)	3.00 (2.50–3.50)		0.120			
**Hospitalization**							
Y	9 (50%)	27 (100%)		**<0.001**			
N	9 (50%)	0 (0%)					
**LOS**	0 (0–1)	1 (1–2)		**0.0001**			

Statistically significant *p*-values are in bold. IQR: interquartile range; PPR: postoperative pain at rest; PPD: Postoperative Pain During Movement; LOS: Length of Stay.

**Table 4:** QoL data.

		Groups		*p*-Value
		BuGs Group (n = 18)	Control Group (n = 27)	
**SF-36**	Physical Functioning	64.45 (56.96–70.13)	66.05 (61.97–72.41)	0.122
	Role Physical	61.73 (57.29–69.01)	67.04 (58.74–77.58)	0.075
	Bodily Pain	68.39 (53.78–72.17)	67.04 (57.95–73.35)	0.391
	General Health	66.70 (56.02–72.79)	66.83 (54.70–72.44)	0.832
	Vitality	67.26 (58.70–71.64)	67.51 (60.02–76.01)	0.334
	Social Functioning	64.74 (54.30–72.46)	65.26 (58.08–72.97)	0.465
	Role Emotional	69.72 (56.20–78.91)	71.20(67.05–78.13)	0.188
	Mental Health	62.23 (51.95–71.11)	66.21 (60.77–73.32)	0.072
**PSQ-18**		4.06 (3.32–4.41)	4.01 (3.61–4.38)	0.871
**TSQ, (IQR)**		43.00 (32.25–53.00)	-	N.A.
**>40 score n (%)**		12 (63.16%)	-	N.A.

Continuous variables are reported as median and IQR. Categorical variables are reported as frequencies and percentage. IQR: interquartile range; SF-36: Short-Form-36; PSQ-18: Short-Form Patient Satisfaction Questionnaire; TSQ: Telemedicine Satisfaction Questionnaire.

## Data Availability

The raw data supporting the conclusions of this article will be made available by the authors on request.

## Abbreviations

The following abbreviations are used in this manuscript:

ASA	American Society of Anesthesiologists
BC	Breast Cancer
BCS	Breast Conserving Surgery
BMI	Body Mass Index
BuGS Trial	Breast Green Surgery Trial
CCI	Charlson Comorbidity Index
CMW	Contaminated Medical Waste
CET	Comitato Etico Territoriale (Territorial Ethical Committee)
CONSORT	Consolidated Standards of Reporting Trials
EBC	Early Breast Cancer
EPT	Estrogen–Progestin Therapy
ERAS	Enhanced Recovery After Surgery
EROM	Environment Reported Outcome Measures
GHG	Greenhouse Gas
Hr	Hour
LOS	Length of Stay
MAC	Monitored Anesthesia Care
MW	Medical Waste
NCMW	Non-Contaminated Medical Waste
NPRS	Numeric Pain Rating Scale
PaMW	Paper Medical Waste
Pl	Plastic Medical Waste
PF	Physical Functioning
PPR	Postoperative Pain at Rest
PPD	Postoperative Pain During Movement
PROM	Patients’ Reported Outcome Measures
PSQ-18	Short-Form Patient Satisfaction Questionnaire
QoL	Quality of Life
RCT	Randomized Clinical Trial
SF	Short-Form
SoC	Standard of Care
TSQ	Telemedicine Satisfaction Questionnaire
TIVA	Total Intravenous Anesthesia
VGA	Volatile General Anesthesia

## Appendix A

**BuGS Study Group:** Nicola Di Lorenzo ^10^, Alice Bertolo ^1^, Alessandra Vittoria Granai ^1^, Adriano De Majo ^1^, Annalisa Noce ^11.^, Valeria Liberto ^12^, Rosaria Meucci ^12^, Tommaso Perretta ^12^, Ilaria Portarena ^13^, Chiara Adriana Pistolese ^12^, Francesca Servadei ^14^, Erica Giacobbi ^14^, and Mario Dauri ^15^.

## Appendix B

**Table A1:** Green Breast Surgery protocol checklist and control group.

Item	BuGS Group	Control Group
Preadmission		
Preadmission information, education, and counseling	Preoperative and detailed preoperative counseling.	Preoperative and detailed preoperative counseling.
Preadmission information, participants’ and hospital optimization	For daily smokers, 1 mo of abstinence prior to surgery is beneficial.	For daily smokers, 1 mo of abstinence prior to surgery is beneficial.
	For obese patients, weight reduction to achieve a BMI ≤ 30 kg/m^2^ prior to surgery is beneficial.	For obese patients, weight reduction to achieve a BMI ≤ 30 kg/m^2^ prior to surgery is beneficial.
	For alcohol abusers, 1 mo of abstinence prior to surgery is beneficial.	For alcohol abusers, 1 mo of abstinence prior to surgery is beneficial.
	For specific patients, referral should be made to resources that can support these behavior changes.	For specific patients, referral should be made to resources that can support these behavior changes.
	Surgical room schedule optimization to avoid unnecessary hospitalization.	Surgical room schedule optimization.
	Outpatient examination.	Preoperative admitted patient examination.
**Preoperative**		
Admission	Admission of the participants in Breast Unit Outpatient clinic.	Hospitalization.
	Hospital staff should not wear non-sterile gloves to move patients unless they come in contact with mucous membranes, biological fluids, or wounds.	Not clearly stated.
	Reduce energy consumption, air conditioning, and lighting in unused rooms.	Reduce energy consumption, air conditioning, and lighting in unused rooms.
Preoperative fasting	Preoperative fasting should be minimized, and patients should be allowed to drink clear fluids up to 2 h prior to surgery.	Preoperative fasting from midnight regardless of the surgical schedule.
Preoperative carbohydrate loading	Preoperative maltodextrin-based drinks should be given to patients 2 h prior to surgery.	None.
Venous thromboembolism prophylaxis	Participants should be assessed for venous thromboembolism risk. Unless contraindicated, and balanced by the risk of bleeding; patients at a higher risk should receive low-molecular-weight heparin or unfractionated heparin until ambulatory. Mechanical methods should be added.	Participants should be assessed for venous thromboembolism risk. Unless contraindicated, and balanced by the risk of bleeding; patients at a higher risk should receive low-molecular-weight heparin or unfractionated heparin until ambulatory. Mechanical methods should be added.
**Intraoperative**		
Antimicrobial prophilaxis	Clorexidine skin preparation should be performed. Intravenous antibiotics should be given within 1 h prior to surgery if needed (e.g., contaminate surgery, postoperative surgical drain, frail patients).	Clorexidine skin preparation should be performed. Intravenous antibiotics should be given within 1 h prior to surgery if needed (e.g., contaminate surgery, postoperative surgical drain, frail patients).
Postoperative nausea and vomiting prophylaxis	Participants should receive preoperative and intraoperative medications to mitigate postoperative nausea and vomiting.	Participants should receive preoperative and intraoperative medications to mitigate postoperative nausea and vomiting.
Preoperative and intraoperative analgesia	Participants should receive multimodal analgesia to mitigate pain.	Participants should receive multimodal analgesia to mitigate pain.
	Participants should receive preoperative locoregional inter-fascial plane blocks, if clinically safe.	According to the anesthesiologist’s preference.
Preparing for surgery	Reusable textiles.	Reusable textiles.
	Reduce water and energy consumption.	Reduce water and energy consumption.
	Reduce invasive intervention (e.g., antibiotics, surgical drains, catheter).	No clear indication.
Standard anesthesia protocol	Local anesthesia or awake multimodal protocol as an opioid-free MAC where local anesthesia, inter-fascial plane blocks, with or without mild sedation, with target RAS > −3 and <1, without use of airway management devices.	According to the anesthesiologist’s preference.
	Targeted O_2_ delivery only if necessary.	According to the anesthesiologist’s preference.
	Minimize drug waste (“Don’t open it unless you need it”, pre-empt propofol use).	According to the anesthesiologist’s preference.
Preventing intraoperative hypothermia	Preoperative and intraoperative measures, such as forced air, to prevent hypothermia should be instituted. Temperature monitoring is required to ensure the patient’s body temperature is maintained above 36 °C.	According to the anesthesiologist’s preference.
Perioperative intravenous fluid management	Overresuscitation or underresuscitation of fluids should be avoided, and water and electrolyte balance should be maintained. Goal-directed therapy is a useful method of achieving these goals. Balanced crystalloid solutions are recommended. Vasopressors are recommended to support fluid management.	According to the anesthesiologist’s preference.
Intraoperative personnel and equipment	Monitor the number of staff in the room.	Monitor the number of staff in the room.
	Create an instrument preference list for each operation: separate essential vs. optional items to have ready ad hoc.	According to the surgeon’s preference.
	Reduce single-use surgical instruments and integrated reusable items.	According to the surgeon’s preference.
	Minimize item waste (“Don’t open it unless you need it”).	According to the surgeon’s preference.
	Hospital staff should not wear non-sterile gloves to move patients unless they come in contact with mucous membranes, biological fluids, or wounds.	According to the surgeon’s preference.
Waste segregation	Recycle or use lowest carbon waste streams as appropriate (use domestic or recycling waste streams for all packaging).	SoC.
	Ensure only appropriate contents in sharps bins (sharps/drugs).	SoC.
	Arrange metals/battery collection where possible.	SoC.
**Postoperative**		
Postoperative analgesia	Multimodal postoperative pain management regimens are opioid-sparing and should be used with locoregional peripheral nerve block, if not already performed in the preoperative setting.	According to the anesthesiologist’s preference.
Early feeding	Patients should be encouraged to intake fluids and food orally as soon as possible, preferably within 3 h after surgery if awake surgery protocol was maintained.	According to the anesthesiologist’s preference.
Postoperative wound management	For incisional closure, conventional sutures are recommended. Complex wounds following skin necrosis are treatable with debridement and negative pressure wound therapy.	According to the surgeon’s preference.
**Postoperative**		
Postoperative analgesia	Multimodal postoperative pain management regimens are opioid-sparing and should be used with locoregional peripheral nerve block, if not already performed in the preoperative setting.	According to the anesthesiologist’s preference.
Early feeding	Patients should be encouraged to intake fluids and food orally as soon as possible, preferably within 3 h after surgery if awake surgery protocol was maintained.	According to the anesthesiologist’s preference.
Postoperative wound management	For incisional closure, conventional sutures are recommended. Complex wounds following skin necrosis are treatable with debridement and negative pressure wound therapy.	According to the surgeon’s preference.
Early mobilization and discharge	Patients should be mobilized within 2 h after surgery. Early physiotherapy, supervised exercise programs, and other supportive care initiatives should be instituted after discharge.	According to the surgeon’s and anesthesiologist’s preference.
	Manage in the outpatient clinic and discharge after 3 h from surgery.	Hospitalization.
Post-discharge home support and physiotherapy	Encourage telehealth and admission to GP for postoperative care.	Hospital admission.

BMI: Body Mass Index; mo: month; h: hour, MAC: monitored care anesthesia; RAS: Richmond agitation–sedation scale; SoC: standard of care; GP: general practitioner.
